# What makes an ideal hospital-based medical leader? Three views of healthcare professionals and managers: A case study

**DOI:** 10.1371/journal.pone.0218095

**Published:** 2019-06-11

**Authors:** Merlijn C. P. van de Riet, Mathilde A. Berghout, Martina Buljac-Samardžić, Job van Exel, Carina G. J. M. Hilders

**Affiliations:** 1 Erasmus School of Health Policy and Management, Erasmus University Rotterdam, Rotterdam, The Netherlands; 2 Erasmus School of Economics, Erasmus University Rotterdam, Rotterdam, The Netherlands; Nord University, NORWAY

## Abstract

Medical leadership is an increasingly important aspect of hospital management. By engaging physicians in leadership roles, hospitals aim to improve their clinical and financial performances. Research has revealed numerous factors that are regarded as necessary for ‘medical leaders’ to master, however we lack insights into their relative importance. This study investigates the views of healthcare professionals and managers on what they consider the most important factors for medical leadership. Physicians (n = 11), nurses (n = 10), laboratory technicians (n = 4) and managers (n = 14) were interviewed using Q methodology. Participants ranked 34 statements on factors elicited from the scientific literature, including personal features, context-specific features, activities and roles. By-person factor analysis revealed three distinct views of medical leadership. The first view represents a *strategic leader* who prioritizes the interests of the hospital by participating in hospital strategy and decision making. The second view describes a *social leader* with strong collaboration and communication skills. The third view reflects an *accepted leader* among peers that is guided by a clear job description. Despite these differences, all respondents agreed upon the importance of personal skills in collaboration and communication, and having integrity and a clear vision. We find no differences in views related to particular healthcare professionals, managers, or departments as all views were defined by a mixture of departments and participants. The findings contribute to increased calls from both practice and literature to increase conceptual clarity by eliciting the relative importance of medical leadership-related factors. Hospitals that wish to increase the engagement of physicians in improving clinical and financial performances through medical leadership should focus on selecting and developing leaders who are strong strategists, socially skilled and accepted by clinical peers.

## Introduction

Medical leadership is an increasingly important topic in both literature and practice, because of the anticipated positive effect that physicians in leadership positions have on quality of care, patient safety and cost efficiency [1–4]. Research shows that hospitals perform better when led by physicians [5–8]. Moreover, physicians are said to have more influence over clinical peers in contrast to non-clinical hospital professionals [4, 9–12]. By engaging in leadership roles, physicians could play an important role in encouraging fellow clinicians in achieving contemporary clinical and organizational objectives.

The importance of and need for medical leadership is reflected in both practice and the literature. Internationally, medical curricula are increasingly adjusting their programs to include leadership competencies, for example the well-known CanMEDS model [13]. Medical students would like more management and leadership training at medical school as now they feel partially unprepared for a future career that is moving beyond clinical boundaries [1, 14–18]. Likewise, educational institutes increasingly offer medical leadership development programs to medical specialists, which physicians value highly [19]. The popularity of medical leadership is also reflected in the scientific literature. The amount of research on the subject is rapidly increasing, mainly yielding insights into the factors, e.g. skills, knowledge, institutional characteristics, activities, that are required for the development of medical leaders and leadership [20–27]. Although both practice and research embrace and plead for the development of medical leadership, there remains a lack of conceptual clarity on the relative importance of factors associated with effective leadership [8, 28].

In response to the fast-growing but scattered literature on what effective medical leadership entails and the skills and knowledge medical leaders should possess, Berghout et al. [28] conducted a systematic literature review. This revealed two broad definitions: a formal managerial role, with a specific appointment, and an informal role, where leadership is inherently part of physicians’ daily work. Irrespective of whether the role is formal or informal, the review elicited three main areas of factors that medical leaders should master: personal features, context-specific features, and activities and roles [28]. Personal features concern the skills, attitude, knowledge, experience in management and credibility a medical leader should have, and include a wide range of character traits, such as communication skills, motivation and clinical knowledge. Secondly, context-specific features refer to management experience, role ambiguity, support and time, and include a variety of institutional and cultural characteristics of the hospital where a medical leader works related to an assumed dichotomy between the managerial and medical world. Finally, the third area consists of the activities and roles required to carry out the role of medical leader, such as strategy and decision making, networking and responsibility for the performance of the department. The long and diverse list of factors is in line with the outcomes of similar literature reviews on medical leadership [14, 29, 30]. This raises the question to what extent a medical leader could, or should, master all the factors and thus of their relative importance.

The diversity of factors could be explained by the potential views of various professionals (e.g. physicians, nurses, laboratory technicians and managers) on what is most important for effective medical leadership. The aim of this study is to provide insight into what healthcare professionals and managers in a specific hospital think is important for effective hospital-based medical leadership. The results of this study could help hospitals to reflect on what type of leadership they aspire to in comparison to the kind of medical leaders they currently have in place. Moreover, hospitals and current medical leaders could use the findings to gain insight into necessary or desirable improvements to enhance the effectiveness of medical leadership. This study could contribute to the development of future medical leaders, by incorporating the factors that are considered important in medical curricula.

## Methods

Q methodology was used to explore healthcare professionals and managers’ views on factors in the areas of personal features, context-specific features, and activities and roles that are thought of as most important for effective medical leadership in a hospital setting. Q methodology combines qualitative and quantitative research techniques to provide a foundation for the systematic study of subjectivity, such as a person’s view or opinion [31, 32]. In conducting a Q-study, researchers present respondents with a comprehensive set of statements about the subject of study, which they are asked to rank according to their view on the subject, and to explain their ranking. By-person factor analysis is used to identify subgroups of respondents who rank the statements in a similar way, resulting in a limited number of distinct composite rankings that can be interpreted and described as the principal shared views on the subject of study. Finally, the qualitative data elicited from the interviews, during which respondents explain their ranking of the most and least important statements, is used to check and refine the interpretation of the quantitative data, and to enrich the description with citations.

This study fell outside the scope of the Netherlands' Medical Research Involving Human Subjects Act (WMO) and therefore no formal ethical approval was needed. Although our research was conducted in a medical setting, it met none of the WMO criteria (http://www.ccmo.nl/en/your-research-does-it-fall-under-thewmo). First, no patients were involved. Second, the study content and methodology did not constitute an infringement of the physical and/or psychological integrity of the participants. This study was part of an overarching research project on medical leadership, which was evaluated by the IRB who confirmed that no ethical approval was required (MEC-2017-409).

### Statement set

The respondents ranked a set of 34 statements about medical leadership. This set derived from an initial set of 37 statements culled from a systematic review of the literature about medical leadership in a hospital setting [28] that provided an overview of factors held important, categorized into three main areas: personal features, context-specific features, and activities and roles. All statements were based on the systematic review of Berghout et al. [28] and additional literature on medical leadership [14, 29, 30] revealed no further factors that would have complemented the statement set. To test the comprehensiveness of the set and the comprehensibility of the statements, a pilot study was conducted among six healthcare professionals (two medical students, a nurse and two physicians) and one quality manager. The total set was reviewed and eventually 11 statements were revised: four statements were combined into two statements because they addressed the same factor, two statements were removed because they were considered irrelevant to the study context, one statement was split into two statements as it addressed potentially separate factors, and four statements were reformulated for clarity. The final set of 34 statements used in the interviews appropriately represented the scientific literature on medical leadership (Table 1).

**Table 1:** Statement set including 34 statements on effective medical leadership (derived from Berghout et al. [28]).

Area	Dimension	Statements
**Personal features**	Skills	1. Have good communication skills
		2. Be able to enthuse and motivate others
		3. Be able to resolve conflicts
		4. Have the skills to manage a team
		5. Have the skills to manage a department
		6. Be able to collaborate
		7. Have good negotiation skills
	Attitude	8. Be assertive
		9. Be a team player
		10. Have integrity
		11. Have an eye for quality and costs and the balance between them
		12. Have a clear vision and be able to convey it to others
		13. Be patient centered
	Knowledge	14. Be excellent in their medical discipline
		15. Knowledge of hospital finances
		16. Knowledge of the structure and processes of the hospital
		17. Knowledge of the Dutch healthcare system
	Experience in management	18. Have experience in leadership
		19. Be trained in leadership
	Credibility	20. Be held in high esteem by fellow physicians
		21. Consider themselves primarily a physician
		22. Being a practicing physician
**Context-specific features**	Competing logics	23. Able to connect the clinical and the management domains
		24. Focus on the interests of the hospital as a whole
		25. Focus on the interests of the clinical departments
	Role ambiguity	26. Have a clear job description of medical leadership
	Support	27. Be accepted as a medical leader
	Time	28. Have sufficient time to execute the leadership role and all associated tasks
**Activities and roles**		29. Be involved in strategy development at the hospital level
		30. Be responsible for the performance of the employees in their department
		31. Be able to initiate improvements
		32. Network and make alliances outside the hospital
		33. Be responsible for the performance of their department
		34. Be able to initiate and maintain cross-department collaborations

### Respondents

In a Q methodology study, participants are selected purposively in order to maximize the possibility of discovering a diversity of views on the subject of study [31]. Since the aim is to explore different views on a specific subject and not the prevalence of these views in the larger population, a relatively small sample is sufficient [31].

The study was conducted at a general district hospital in the Netherlands. This hospital is committed to developing medical leadership and therefore offers a leadership program for physicians. As this hospital has been intrinsically interested in medical leadership for 10 years at least, it offered a useful setting to study the matter. It was possible to gain access to three departments and obtain the cooperation of all kinds of healthcare professionals and managers. Although the study was conducted in only one hospital, the ability to include such a variety of departments and professionals enabled this study to provide a broad representation of views on medical leadership. A total of 39 healthcare professionals and managers from the departments of radiology, internal medicine and surgery were asked to participate. These departments were selected because they represent three large overarching units committed to different types of care delivery. Therefore, the healthcare professionals and managers working at these departments were expected to represent a variety of views on what is important for medical leadership across the broader hospital setting. To maximize the possibility of finding the principal views on this subject, four kinds of professionals were interviewed: managers (n = 14), physicians (n = 11), and nurses (n = 10) or laboratory technicians (n = 4) (Table 2). Managers include six business managers and seven team heads, who hold a background in nursing. The category of ‘physicians’ also includes five physicians who manage their clinical departments part-time. As the department of radiology does not employ nurses, laboratory technicians were asked to participate. The professionals were approached by the secretary of each department and selected on availability.

**Table 2:** Background characteristics of total sample.

Characteristic	Surgical (n = 13)	Radiology (n = 13)	Internal medicine (n = 13)	Total (n = 39)
**Sex (% female)**	69%	46%	69%	62%
**Mean age (years)**	43	44	44	44
**Profession**				
** Manager**	4	5	5	14
** Physician**	4	4	3	11
** Nurse**	5	0	5	10
** Laboratory technician**	0	4	0	4
**Fulltime/part-time (% fulltime)**	86%	86%	71%	81%
**Mean years employed in current function**	7	9	6	7
**Mean years employed in this hospital**	13	16	14	14
**Management school or cursus (% yes)**	62%	64%	46%	57%

### Data collection

Quantitative and qualitative data were collected through semi-structured interviews, during which the respondents ranked the 34 statements according to importance for medical leadership. On finishing the ranking exercise, they answered questions to clarify their ranking. All interviews were conducted in April 2017 and lasted between 20 to 45 minutes. To ensure that the respondents were well informed, medical leadership was first briefly defined: a medical leader is always a physician, but the role of a medical leader can be either formal or informal.

**Fig 1:**
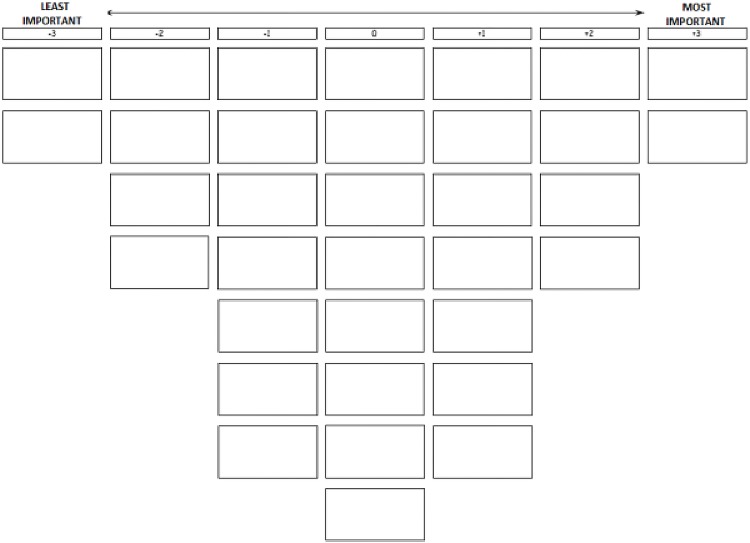
Score sheet.

After the introduction, respondents were handed the statement set, printed on cards and randomly ordered, and a sorting grid (Fig 1). The respondents were asked to read all the statements and divide them into three piles: (1) important, (2) neutral and (3) unimportant for effective medical leadership. Next, the respondents were instructed to read all the statements from each pile again, consecutively, and to rank them on the grid. After finishing the ranking exercise, respondents were asked to check their ranking by reading all statements again and adjust the ranking, if needed. Subsequently, the respondents were asked to explain the placement of the statements in the most important and least important columns. All respondents were able to sort their statements into the shape of the Q sort shown in Fig 1. At the end of the interview, respondents answered questions about a number of background characteristics, which are listed in Table 2. Finally, the respondents were asked whether they felt the statement set was complete or if there were any factors lacking. A minority of the respondents (N = 14) mentioned approachability of a medical leader, remuneration for the time a medical leader spends on additional tasks and listening to others as factors related to medical leadership. During the initial development of the statement set these factors were not included as they were not or scarcely mentioned in literature [28], neither were they mentioned during the pilot study. All interviews were recorded and transcribed with the permission of the respondent.

### Analysis

To analyze the rankings, we used PQMethode 2.11 software [33]. We conducted by-person factor analysis with centroid factor extraction and varimax rotation, resulting in a three-factor solution that explained 44% of the variance in ranking data. An idealized ranking of 34 statements was computed, based on the rankings of the respondents that were associated with that factor (Table 3). These idealized rankings were interpreted as distinct views on what is important for effective medical leadership, focusing on the statements that characterize each of the factors (i.e., those ranked in the outer columns of the idealized ranking of the factor) and those that distinguish between factors (i.e., with a statistically significantly different rank score in a factor as compared to the other factors). The first interpretations of the three factors based on the quantitative data were further refined using the qualitative data retrieved from the semi-structured interviews. Some explanations from respondents associated with a factor are cited for illustration of the interpretation of that factor.

**Table 3:** Idealized ranking per view of the 34 statements on effective medical leadership for the full sample.

Statements	View 1 Strategic leader	View 2 Social leader	View 3 Accepted leader
**Personal features**			
**1. Have good communication skills**	1**	3*	2*
**2. Be able to enthuse and motivate others**	2	1	3
**3. Be able to resolve conflicts**	1	2**	0
**4. Have the skills to manage a team**	0*	1	1
**5. Have the skills to manage a department**	-1*	3**	0*
**6. Be able to collaborate**	1**	3	2
**7. Have good negotiation skills**	-1	1**	0
**8. Be assertive**	0**	1**	-1**
**9. Be a team player**	0**	1	1
**10. Have integrity**	2	2	2
**11. Have an eye for quality and costs and the balance between them**	1**	0	0
**12. Have a clear vision and be able to convey it to others**	3**	2	2
**13. Be patient centered**	3*	2	1
**14. Be excellent in their medical discipline**	-1	-1	-3**
**15. Knowledge of hospital finances**	0	-1	-2**
**16. Knowledge of the structure and processes of the hospital**	0	0	1**
**17. Knowledge of the Dutch healthcare system**	-1*	-2**	0*
**18. Have experience in leadership**	-2	0**	-2
**19. Be trained in leadership**	-1	1**	-1
**20. Be held in high esteem by fellow physicians**	-2	-3	-2
**21. Consider themselves primarily a physician**	-3	-2	-1**
**22. Be a practicing physician**	-1**	-2**	1**
**Context-specific features**			
**23. Be able to connect the clinical and the management domains**	2*	0	1
**24. Focus on the interests of the hospital as a whole**	2**	0**	-2**
**25. Focus on the interests of the clinical departments**	-2	0	-1
**26. Have a clear job description of medical leadership**	-2**	-1**	3**
**27. Be accepted as a medical leader**	-1**	-2**	3**
**28. Have sufficient time to execute the leadership role and all associated tasks**	1	0	1*
**Activities and roles**			
**29. Be involved in strategy development at the hospital level**	3**	-1	-1
**30. Be responsible for the performance of the employees in his/her department**	0	-1	-1
**31. Be able to initiate improvements**	1	1	0**
**32. Network and make alliances outside the hospital**	0*	-1	-1
**33. Be responsible for the performance of their department**	0	0	0
**34. Be able to initiate and maintain cross-department collaborations**	1**	-1**	0**

** p < .01,

* p < .05. Scores range between -3 and +3 (Fig 1)

## Results

Thirty-nine respondents participated in the study, 13 from each of the departments (Table 2). The analysis revealed three main shared views on what is important for effective medical leadership in a hospital setting, with 36 (92%) respondents associating statistically significantly with one of the factors. The views are described below with reference to the placement of statements in the idealized ranking of the factor (Table 3).

### View 1: The strategic leader

This view contains two main aspects that the respondents find important for a medical leader. The first aspect relates to the respondents’ concerns about lack of unity in the hospital. Respondents argued that every department favors its own interests over hospital-broad objectives resulting in a culture of ‘conflicting islands’ that in turn could jeopardize the quality of care delivery. Therefore, they want a medical leader capable of forging unity between and beyond clinical departments by participating in hospital strategy and decision making [statement (st.) 29, scored as +3**]. According to the respondents, a medical leader needs to transcend professional boundaries, connect the clinical and management domains (st. 23, 2) and pursue the interests of the hospital instead of merely the departments (st. 25, +2).

*“After the hospital renovations*, *clinical departments turned into little islands*, *not hearing or seeing each other anymore*. *That used to be different*. *[…] We don’t help each other out so much anymore nor do we know what is happening in other clinical departments*. *[…] I think this comes at the expense of being a unity as a hospital*.*”*

(Surgery nurse 2)

Respondents argued that improving unity within the hospital and increasing collaboration between healthcare professionals and non-clinicians (e.g. managers, support staff, and directors) would eventually have a positive effect on the clinical departments too. Having a clear vision for a clinical department that is in line with the hospital’s strategy and being able to convey it to others was therefore ranked as most important (st. 12, +3**) (st. 24, +2**). In this view the patient should always be the main priority (st. 13, +3*), although many respondents argued that this should be obvious and thus unnecessary to mention explicitly.

*“I think that as a medical leader you should be involved in decision making and determining the strategy of the hospital*. *Only then can you defend and convey these choices to your department*, *which in turn is only possible when you have taken part in these discussions*.*”*

(Internal medicine manager 3)

The second aspect deemed important for medical leadership in this view is the importance of having strong personal skills. Most of all, a medical leader should have integrity (st. 10, +2) and be able to motivate and enthuse others (st. 2, +2). Integrity was perceived as important for gaining trust and respect. Although not ranked as the relatively most important, communication and collaboration skills were often underscored as necessary to create unity and engage others in executing their vision (st. 1, +1**) (st. 6, +1**). In contrast, the respondents ranked “be held in high esteem by fellow physicians” (st. 20, -2) as relatively less important for effective medical leadership. Respondents stated that popularity does not immediately turn someone into a good medical leader, while integrity was argued as essential for medical leaders to get things done:

*“For example*, *look at Mark Rutte (Dutch prime minister)*. *I don’t think everyone admires him*, *but I think he’s a good leader*. *So*, *respect is not connected to how you do your work*. *That a medical leader brings in important things for the patient or for the hospital is more important than how popular that medical leader is at work*.*”*

(Surgery nurse 4)

Respondents perceived training (st. 19, -1), work experience (st. 18, -2) and a clear job description (st. 26, -2**) as relatively unimportant for medical leadership. Respondents argued that being a medical leader is either part of your personality or not and can therefore not be taught. They stated that education or job descriptions could support a medical leader, but that strong personal skills such as being able to convey a vision to others are more important for a medical leader to possess.

Additionally, professional identity in terms of a medical leader considering themselves primarily a physician (st. 21, -3) was ranked relatively unimportant for effective medical leadership. Participants felt that leadership activities and clinical work are equally important. Some even questioned whether a medical leader has to be a physician or if other healthcare professionals can execute leadership roles as well:

“*A medical leader can also be someone who has not necessarily specialized as a physician*.”

(Surgical nurse 2)

In conclusion, this view represents a strategic leader, who prioritizes the interests of the hospital over clinical department-specific interests by participating in hospital strategy and decision making. A strategic leader has a personality that reflects integrity and is not subject to status or experience. This view is represented by managers (n = 8), physicians (n = 5), nurses (n = 3) and laboratory technicians (n = 1) and explained 21% of the variance in rankings.

### View 2: The social leader

The second view represents a social leader. Holders of this view regard personal skills, specifically strong communication and collaboration skills, as most important for medical leadership (st. 1, +3*) (st. 6, +3). Respondents argued that these skills enable a medical leader to manage a department effectively (st. 5, +3**) and resolve conflicts (st. 3, +2**) among department members. Moreover, by participants considered communication skills and the ability to collaborate necessary to convey a clear vision to others (st. 12, +2) and to engage others in executing their vision:

*“Decision making must be transparent to all*. *Occasionally you have to explain very clearly why you are making a certain decision*, *because then people will be more likely to follow you*, *not always*, *but far more*.*”*

(Surgery physician 1)

Respondents explained, however, that in terms of a formal type of medical leadership, medical managers were often not chosen for these skills but for more practical reasons, such as their availability or motivation. Consequently, medical department leaders did not always have social skills. Respondents argued that having strong ties with all the staff and knowing “what’s going on” is important in preventing friction among staff and improving decision making.

The holders of this second view implied that it is more important for a medical leader to possess leadership skills than medical excellence or being a practicing physician. Respondents argued that medical leadership can only be effective when physicians fully commit to the responsibility of being a leader. In contrast to the first view—that a medical leader should balance between being a medical leader and a physician—the second view reflects a medical leader who does not consider themselves primarily a physician (st. 21, -2). According to the respondents, being a practicing physician may even stand in the way of being a good medical leader (st. 22, -2**) as it could increase the chance of favoring clinical issues, which might not benefit long-term objectives. Likewise, acceptance and being held in high esteem were considered less important in this view, as respondents believed that neither one is a premise for effective medical leadership (st. 20, -3) (st. 27, -2**):

*“I think that being held in high esteem by fellow physicians has little to do with whether you are a good medical leader or not*. *There are also physicians who are held less high in esteem*, *but are very good at managing*.*”*

(Surgery physician 3)

Having a medical background, however was considered a prerequisite for being a medical leader or head of a clinical department as is enables the leader to correctly interpret issues and set the right goals for the department. Yet, the respondents argued that a medical leader does not need to master specific managerial knowledge. For example specific knowledge of the Dutch health system was perceived as least important for effective medical leadership (st. 17, -2**). When a medical leader has strong social skills and can collaborate with others, any financial or policy-related information can easily be obtained if required from (non-clinical) colleagues:

*“I think that basic knowledge is enough*. *A medical leader must of course know something about the system*, *must know something about finance*, *but it’s not the most important thing in the role of medical leader*. *If you have the skills to listen and trust others*, *then you don’t have to have that knowledge yourself*.*”*

(Internal medicine manager 3)

In conclusion, the second view represents a social medical leader, who is known for strong collaboration and communication skills instead of medical excellence. These social skills increase the capability of the medical leader to convey their vision clearly to others. This view is held by managers (n = 3), physicians (n = 3) and laboratory technicians (n = 3) and explained 11% of the rankings variance.

### View 3: The accepted leader

The third view reflects a medical leader who is guided by a clear job description and is accepted by others in their medical leadership role (st. 27, +3**) (st. 26, +3**). Respondents holding this view felt that they, and fellow clinicians, were not always well informed about the tasks and duties of a medical leader. Ambiguity regarding the medical leadership was seen as a cause of occasional confusion and frustration inside a clinical department. Respondents said that having a clear job description of medical leadership allows the medical leader to execute tasks more efficiently and improves expectation management among clinical peers.

Again, respondents emphasized the importance of communication skills (st. 1, 2*) and the ability to enthuse and motivate others (st. 2, 3). These skills were deemed necessary to create clarity about the responsibilities of the medical leader, but even more to keep all staff informed about and engaged in (proposed) changes in department processes and care delivery. In turn, respondents argued that clarity leads to the good working atmosphere that favors staff well-being and the quality of care.

Acceptance of a medical leader was said to be highly important to engage fellow clinicians (physicians and nurses) in future changes or developments. At the same time, respondents argued that physicians play a big role in decision making and therefore peers perceived their credibility as a medical leader as key to effective leadership. Fir a medical leaders to create acceptance among clinical peers, being able to collaborate (st. 6, +2), having a clear vision and being able to convey it to others (st. 12, +2) were considered relatively important:

*“Working together is a core value in healthcare*. *The medical manager happens to be the head of the department*, *but we all have to pull together*. *Otherwise you lack acceptance as a leader … You may want to go in a certain direction*, *but if your colleagues are not behind you*, *it will be hard to convey your vision*.*”*

(Internal medicine manager 2)

Similar to the second view, being held in high esteem by fellow physicians (st. 20, -2) and being excellent in their medical discipline (st. 14, -3**) were not regarded as important factors.

*“I don’t care about respect or peers holding someone in high esteem*. *I personally don’t think it’s important*. *I’d like everyone to treat each other with respect*, *regardless of whether I’m a medical manager or a radiologist*.*”*

(Radiology physician 2)

*“You have to be good at your medical discipline and understand what others are doing*. *Make that you behave well*. *But it doesn’t mean you have to be excellent*. *I think being a leader has a higher priority than being excellent in your discipline*.*”*

(Internal medicine nurse 4)

Likewise, leadership experience (st. 18, -2) was not considered a precondition for effective medical leadership. Rather, holders of this view perceive leadership as an innate characteristic which does not come with experience or education:

“*Some people are just born leaders*. *I think you can function as an informal leader without experience*. *You can learn a lot*, *but you have to have certain personality traits if you want to be a leader*. *You can’t learn it all; some people are no good at it by nature*.*”*

(Internal medicine nurse 2)

In a similar vein respondents argued that a medical leader should not have to possess specific knowledge that can easily be obtained from others, for example, on hospital finance (st. 15, -2**). Rather, a medical leader needs to know where they can find the required knowledge and should be able to establish valuable cooperation with others:

*“The specific knowledge is present in the hospital*, *so that doesn’t mean that you need to know it all yourself straight away*. *You can also check and review things*. *I think that's more like the ability to find the right people in the right place*.*”*

(Radiology manager 5)

Finally, the respondents of this view ranked “focus on the interests of the hospital as whole” (st. 24, -2**) as least important in medical leadership. This finding is in contrast to the first and second views that both made prioritizing hospital objectives as a characteristic of good medical leadership. The respondents of this third view, however, argued that a medical leader should focus on the quality and efficiency of one clinical department first:

“*If your own field does not function properly, the entire hospital cannot function properly either*.”

(Internal medicine nurse 4)

In conclusion, the third view represents a medical leader who is accepted among peers. The proper execution of medical leadership requires a clear job description. This view is represented by managers (n = 2), physicians (n = 2) and nurses (n = 5) and explained 12% of the variance in rankings.

### Differences and similarities between the three views

We observed three remarkable differences between the three views. The first distinction concerns the prioritization of either hospital or department interests. The medical leader in view 1 conveys a hospital-wide vision to overcome fragmentation and increase unity while the medical leader in view 3 prioritizes the interests of their own clinical department, arguing that the performance of individual clinical departments is a premise for the performance of a hospital as a whole. Second, the importance of peer acceptance was perceived differently. In view 3, respondents argued that peer acceptance is key for medical leadership as without acceptance a medical leader would be unable to engage others in executing their vision. In contrast, in view 2 respondents interpreted peer acceptance as a basic principle of collegiality, related to trust and respect, and was not regarded as a guarantee for successful leadership. Third, view 2 states that a medical leader should prioritize the duties of leadership over clinical work, while view 1 values leadership and clinical responsibilities equally. Similarly, being a practicing physician was ranked as relatively unimportant in view 2, whereas respondents in views 1 and 3 were more neutral toward this statement.

All three views ranked personal features as relatively important for medical leadership. Specifically, strong communication skills, collaboration skills, integrity and having a vision and being able to convey this to others were ranked as most important for a medical leader to possess.

There were small differences for other personal features as relatively important. Views 1 and 3 prioritized being able to enthuse and motivate others, while view 2 perceived resolving conflicts and possessing management skills as more important.

With regard to what respondents perceived as relatively unimportant, all interviewees agreed that being held in high esteem by fellow physicians, leadership experience, considering yourself primarily a physician and mastering specific managerial knowledge were the least important factors. All views stated that peer approval, or popularity, does not immediately turn a physician into a good medical leader and was thus not regarded as a premise for medical leadership. Concerning leadership experience, the respondents argued that being a good leader is often an innate part of your character and does not come from years of experience. Considering yourself primarily a physician was perceived as unimportant in all three views. However, the interpretation of the statement differed between the first two views. Whereas view 1 felt that a medical leader should balance between being a leader and a physician, view 2 stated that a medical leader can only be effective when prioritizing leadership-related work. All respondents ranked possessing managerial knowledge as relatively unimportant because this could easily be obtained from (non-clinical) colleagues. Finally, we found no differences in views between different professionals or departments as all views were defined by a mixture of the departments and healthcare professionals and managers.

## Discussion

This study distinguished three views of healthcare professionals and managers on what is most important for medical leadership in a hospital. The first view represents a *strategic leader* who prioritizes the interests of the hospital by participating in hospital strategy and decision making. Holders of this view argue that this type of leadership is needed in hospitals to create more unity between clinicians and non-clinicians in favor of quality and efficiency of care. The second view describes a *social leader* who has strong collaborative and communication skills. Respondents holding this view state that social skills are a premise for efficiently leading a clinical department and engaging all staff in creating a shared vision. The third view reflects an *accepted leader* who is guided by a clear job description. Peer acceptance and clarity concerning the responsibilities of a medical leader were considered necessary to engage fellow staff in decision making and change processes. Despite their differences, all participants agreed upon the importance of personal skills, specifically communication skills, collaboration skills, integrity and having a vision and being able to convey this to others. All interviewees perceived being held in high esteem by fellow physicians, leadership experience, considering yourself primarily a physician and mastering specific managerial knowledge the least important factors for medical leadership.

The findings are in line with previous studies on medical leadership. In specific, scholars often underscore the importance of communication skills [4, 20–23, 26, 27, 34–36] and collaboration skills [20, 22, 24, 25, 34, 36, 37]. Having a clear vision [21, 24, 26, 27, 34] and integrity [21, 23–25] were also emphasized as important in literature, however, mentioned less often. Statements that were ranked as important by participants, but were not statistically significant were nonetheless found in literature to be important: connecting the clinical and management domain [view 1: 4, 11, 22, 34–38] pursuing the interests of the hospital [view 1: 20, 26, 35, 36] and being able to motivate and enthuse others [view 1 and 3: 20–25, 27, 35].

Our findings are furthermore in line with a recent stream of literature that shows that professionals are increasingly engaged in healthcare improvement and organizational issues [12, 18; 39–41]. Adhering to the notion of ‘organized professionalism’, social scientists are moving beyond the assumption that professionalism and managerialism are intrinsically conflicting and argue instead that these can co-exist [39–41, 42, 43, 44]. In a similar vein, medical leadership is seen as a key element in dealing simultaneously with pressures for increasing efficiency and quality of care [8, 44]. Likewise, our findings showed that healthcare professionals and managers support the involvement of physicians, and arguably other healthcare professionals, in leadership roles and managerial activities. The respondents, both clinical professionals and managers, underscored the necessity of transcending clinical and departmental borders [view 1] to stimulate hospital unity and multidisciplinary collaboration between (non) clinicians [view 1, 2 and 3]. To what extent physicians should prioritize leadership-related duties over clinical work was, however, perceived differently by the respondents. View 1 stated that clinical work and medical leadership are of equal importance, while view 2 argued that medical leadership can only be effective when the physician fully commits themselves to the responsibilities of being a leader. View 3 was represented by respondents who were less familiar with medical leadership and therefore perceived clarity about the role as relatively most important.

Previous studies show that being held in high esteem by fellow physicians and identifying as primarily a physician are significant for being a medical leader [4, 11, 20, 34, 35, 38, 45]. Scholars have shown that this is important to prevent peers from interpreting medical leaders as ‘agents of government to control the expert power of the professional’ [46]. Interestingly, the results of our study suggest the opposite, as healthcare professionals and managers rated both features as relatively unimportant. Instead, respondents representing the first view argued that a medical leader should create unity *between physicians and managers* and prioritize hospital-wide objectives over department-specific ones. The second view even argued that a medical leader should fully commit to leadership-related duties to decrease the chance of favoring clinical issues, which not always benefits the long-term objectives. In conclusion, all respondents stated that popularity does not immediately turn someone into a good medical leader, while integrity (view 1), prioritization of leadership (view 2) and acceptance (3) were argued as necessary for medical leaders in order to get things done. Although these findings are contrary to previous studies on medical leadership and management, recent studies on the topic show similar results. Studies among both mid-career physicians and medical students show the increasing willingness of physicians to engage in healthcare improvement and organizational issues through medical leadership [12, 17, 18, 44, 47–50].

The final remarkable outcome of our study was the low ranking of facilitating factors such as leadership experience and training. Previous research has, however, extensively described the need for training and experience in medical leadership among medical physicians and students [1, 28, 36]. Current physicians in leadership argue that lack of training and experience lead to insecurity, stress, and frustration and hinder them from performing their role effectively [28, 37, 45, 51]. Likewise, medical students advocate for the incorporation of leadership and management training in medical curricula as they feel that their current training program does not prepare them properly for their future medical careers [1, 16, 17, 48]. Our distinctive finding could be explained by the fact that our respondents argued that effective medical leadership is innate and depends on a person’s character and not on experience and training. Important to note here is the fact that these features were ranked least important in this study does not mean that the respondents felt they had no value whatsoever. The design of the study required the respondents to arrange the factors in order of relative importance.

We found no differences in views between different professionals or departments as all views were defined by a mixture of the departments, healthcare professionals and managers. Based on our findings, we suggest that what healthcare professionals and managers deem important for medical leadership is not determined by their professional background or specialism. However, most of the nurses we interviewed argued that medical leaders do not have to be a physician per se, as long as they have a medical background. This argument is underlined by several scholars, who plead for nursing leadership by showing its importance [52–54] and the ability of nurses to fulfill similar leadership roles [55].

### Limitations

This study has four limitations. First, the set of statements was developed from English literature reviews and translated into Dutch. A previous study showed that English and Dutch speakers vary in how strongly they use various syntactic cues to interpret sentences, like prepositions or word order [56]. Therefore it can be argued that statements could be interpreted differently in various settings, for example “be held in high esteem by fellow physicians” and “be accepted as a medical leader”. However, we believe that this limitation is restricted as the pilot study did not show reason for doubt. Second, the perceptions of medical leadership can be influenced by the short introduction to medical leadership before the ranking. The role of medical leader was explained in two ways, as an informal or a formal role. As the clinical departments of the hospital where our inquiry took place are guided by medical managers (formal medical leadership role) it is not clear whether the respondents interpreted medical leaders as formal leaders, their medical manager, or informal leaders, which could be any physician. Third, our sample came from three departments of one general hospital that is already focused on medical leadership and provides training in medical leadership, which possibly makes our findings not generalizable to other clinical departments or hospitals. Although we found no specific differences in views on medical leadership among the three clinical departments, it could be that certain departments encounter different medical and organizational issues that ask for a different medical leader as suggested in a study by Meretoja et al. [57]. We thus recommend replication of this study among healthcare professionals and managers in different settings to confirm if these views are applicable to other clinical departments (e.g. gynecology, oncology), types of hospitals (e.g. teaching hospitals) or even other countries. Finally, during the data collection the respondents were asked whether they felt the statement set was complete or if there were any factors lacking. A minority of the respondents mentioned a few additional factors, which they linked to medical leadership: approachability of a medical leader, remuneration for the time a medical leader spends on additional tasks, and listening to others. During the initial development of the statement set we did not include these factors as they were not or scarcely mentioned in literature [28], neither were they mentioned during the pilot study. We do not claim that the statement set includes all factors related to medical leadership. The factors most often mentioned in literature are however represented in the statement set.

### Implications

Our findings translate into one scientific and two practical implications. The scientific implication is that this study increases conceptual clarity about medical leadership by investigating the relative importance of factors that are related to it. We thereby respond to increased callings in literature and practice for more conceptual clarity [1, 28, 45]. Future studies could examine how current or future medical leaders develop themselves as one, or a mixture, of these types of leaders (strategic, social, or accepted) and how these types of leadership influence quality and efficiency of care. The first practical implication is that our findings can be used to improve medical education and leadership programs. Based on our findings, medical curricula, hospital training for medical managers and leaders and medical leadership development programs should focus more on personal development, specifically communication skills, collaboration skills, having a clear vision and being able to convey it to others, and resolving conflicts. These factors are deemed more important than, for example, merely focusing on financial and management skills or knowledge of healthcare systems [58, 59] This outcome is also emphasized in the well-known CanMEDS framework that recently replaced the physician’s core value ‘manager’ with ‘leader’ [59]. The second practical implication is that the results of this study could contribute to the professionalization of recruiting medical managers (or ‘clinical directors’) in hospitals. In selecting medical managers, hospitals should move the focus from physicians who are held in high esteem by peers or are known for their medical excellence, to physicians with strong interpersonal skills in communication and collaboration, and who have a strong vision and are able to convey it to others. This could arguably lead to increased effective medical leadership as the respondents of this study argue that current medical leaders, in this case medical managers, do not always seem ‘fit for the job’.

## Supporting information

S1 File Data. (PDF)

S2 File Statement set and questionnaire. (PDF)
