# Supplementary material for: What makes an ideal hospital-based medical leader? Three views of healthcare professionals and managers: A case study
Source: PLoS One. 2019 Jun 11;14(6):e0218095. doi: 10.1371/journal.pone.0218095 (PMC6559653; doi:10.1371/journal.pone.0218095)
Supplement: S1 File — (PDF) [file pone.0218095.s001.pdf]

S1 File. Data.

3-factor oplossing

Factor Matrix with an X Indicating a Defining Sort

QSORT		Loadings 1	2	3
1	Iarts01	0.0970	0.1441	0.4132X
2	Iarts02	0.6780X	0.2702	0.3279
3	Iarts03	0.6129X	0.0856	0.1714
4	Ivpk01	0.2281	0.2766	0.3491
5	Ivpk02	-0.0239	0.4217	0.4852X
6	Ivpk03	0.5320X	0.1806	0.1212
7	Ivpk04	0.0698	0.2056	0.6457X
8	Ivpk05	0.3584	0.4240	0.5510
9	Imana01	0.3777	0.6805X	0.1340
10	Imana02	0.1204	0.1407	0.6059X
11	Imana03	0.4595X	0.4280	-0.0059
12	Imana04	0.3849	0.5095X	0.2123
13	Imana05	0.7174X	0.1452	0.0522
14	Rlab01	0.5339X	0.3450	0.2638
15	Rarts01	0.3194	0.1852	-0.1100
16	Rarts02	0.2091	0.1689	0.5289X
17	Rman01	0.5621X	-0.0112	0.2405
18	Rarts03	0.7783X	0.0615	0.1065
19	Rman02	0.3425X	0.2035	0.2533
20	Rlab02	0.0998	0.4630X	0.3370
21	Rman03	0.2408	0.6406X	0.0258
22	Rlab03	0.0030	0.6275X	0.0238
23	Rlab04	0.3485	0.5342X	0.3214
24	Rman04	0.5809X	-0.0446	0.2888
25	Rman05	0.3423	0.1329	0.5413X
26	Rarts04	0.6400X	0.2136	0.5000
27	Cmng01	0.7039X	0.2640	0.0827
28	Cmng02	0.5372X	-0.0468	0.3404
29	Cmng03	0.6438X	0.2496	0.2839
30	Cmng04	0.7492X	0.1817	0.1843
31	Cvpk01	0.1313	-0.0483	0.7373X
32	Cvpk02	0.6578X	0.2554	0.2636
33	Cvpk03	0.1422	0.1275	0.5069X
34	Cvpk04	0.7150X	0.2849	0.1146
35	Cvpk05	0.1616	-0.1305	0.3401X
36	Carts01	0.0683	0.4464X	0.1107
37	Carts02	0.2516	0.5546X	0.0689
38	Carts03	0.0259	0.4834X	0.4393
39	Carts04	0.5768X	0.2702	-0.4836
% expl.Var.		21	11	12

Factor Scores with Corresponding Ranks

No.	Statement	No.	Factor 1	Rank	Factor 2	Rank	Factor 3	Rank
1	Goede communicatieve vaardigheden	1	0.61	12	1.72	1	1.14	7
2	In staat zijn om anderen te enthousiasmeren en te moti	2	1.19	5	1.01	8	1.52	1
3	In staat zijn om conflicten op te lossen	3	0.27	14	1.18	4	0.47	15
4	Vaardigheden om een team te managen	4	0.04	15	0.54	11	0.61	11
5	Vaardigheden om een afdeling te managen	5	-0.51	24	1.57	2	-0.10	17
6	In staat zijn om goed samen te werken	6	0.61	11	1.54	3	1.23	4
7	Goede onderhandelingsvaardigheden	7	-0.59	25	0.38	13	-0.27	21
8	Assertiviteit	8	-0.31	19	0.33	14	-1.10	29
9	Een team player zijn	9	-0.08	17	0.77	9	0.60	12
10	Integriteit	10	1.38	4	1.05	6	1.17	6
11	Oog hebben voor de kwaliteit en kosten en de balans hi	11	0.76	10	0.15	18	-0.23	20
12	Een duidelijke visie hebben en in staat zijn deze over	12	1.81	1	1.14	5	1.22	5
13	PatiÎnt centraal stellen	13	1.49	3	1.03	7	0.67	10
14	Excellent zijn in zijn/haar medisch vakgebied	14	-0.65	27	-0.99	28	-2.04	34
15	Kennis over ziekenhuisfinanciÎn	15	-0.42	22	-0.75	24	-1.49	32
16	Kennis over structuur en processen van het ziekenhuis	16	0.02	16	-0.13	20	0.74	9
17	Kennis over het Nederlandse zorgsysteem	17	-0.59	26	-1.44	31	-0.11	18
18	Ervaring in leiderschap	18	-1.47	31	-0.15	21	-1.36	30
19	Getraind of opgeleid in leiderschap	19	-1.18	29	0.51	12	-1.02	28
20	Aanzien bij collega artsen	20	-1.56	32	-1.97	34	-1.67	33
21	Een medisch leider ziet zichzelf voornamelijk als arts	21	-1.68	34	-1.60	33	-0.61	25
22	Werkzaam als behandelend arts	22	-0.85	28	-1.47	32	0.57	13
23	In staat om een verbinding te maken tussen het medisch	23	1.06	7	0.23	16	0.56	14
24	Het belang van het gehele ziekenhuis centraal stellen	24	1.19	6	0.26	15	-1.38	31
25	Het belang van de vakgroep centraal stellen	25	-1.24	30	-0.51	22	-0.91	27
26	Een duidelijke functieomschrijving van medisch leiders	26	-1.62	33	-0.85	26	1.52	2
27	Acceptatie van de arts als medisch leider	27	-0.50	23	-1.11	30	1.23	3
28	Voldoende tijd om leiderschapsrol en bijbehorende take	28	0.32	13	0.18	17	0.77	8
29	Betrokkenheid bij het vormgeven van de strategie van h	29	1.51	2	-0.79	25	-0.37	23
30	Verantwoordelijk voor het functioneren van medewerkers	30	-0.40	21	-0.61	23	-0.61	24
31	Verbeteringen initiÎren	31	0.97	9	0.66	10	-0.15	19
32	Netwerken en verbindingen maken buiten het ziekenhuis	32	-0.35	20	-0.87	27	-0.77	26
33	Verantwoordelijk voor de resultaten van de eenheid waa	33	-0.21	18	0.04	19	-0.31	22
34	Afdelingsoverstijgende samenwerking opzetten en/of in	34	0.98	8	-1.03	29	0.46	16

Correlations Between Factor Scores

	1	2	3
1	1.0000	0.5484	0.4396
2	0.5484	1.0000	0.4299
3	0.4396	0.4299	1.0000

Factor Scores -- For Factor 1

No.	Statement	No.	Z-SCORES
12	Een duidelijke visie hebben en in staat zijn deze over te br	12	1.807
29	Betrokkenheid bij het vormgeven van de strategie van het zie	29	1.514
13	PatiÎnt centraal stellen	13	1.493
10	Integriteit	10	1.382
2	In staat zijn om anderen te enthousiasmeren en te motiveren	2	1.192
24	Het belang van het gehele ziekenhuis centraal stellen	24	1.188
23	In staat om een verbinding te maken tussen het medische -en	23	1.056
34	Afdelingsoverstijgende samenwerking opzetten en/of in stand	34	0.984
31	Verbeteringen initiÎren	31	0.973
11	Oog hebben voor de kwaliteit en kosten en de balans hierin	11	0.762
6	In staat zijn om goed samen te werken	6	0.612
1	Goede communicatieve vaardigheden	1	0.607
28	Voldoende tijd om leiderschapsrol en bijbehorende taken uit	28	0.316
3	In staat zijn om conflicten op te lossen	3	0.270
4	Vaardigheden om een team te managen	4	0.038
16	Kennis over structuur en processen van het ziekenhuis	16	0.025
9	Een team player zijn	9	-0.084
33	Verantwoordelijk voor de resultaten van de eenheid waar leid	33	-0.207
8	Assertiviteit	8	-0.305
32	Netwerken en verbindingen maken buiten het ziekenhuis	32	-0.353
30	Verantwoordelijk voor het functioneren van medewerkers aan w	30	-0.405
15	Kennis over ziekenhuisfinanciÎn	15	-0.420
27	Acceptatie van de arts als medisch leider	27	-0.504
5	Vaardigheden om een afdeling te managen	5	-0.512
7	Goede onderhandelingsvaardigheden	7	-0.590
17	Kennis over het Nederlandse zorgsysteem	17	-0.592
14	Excellent zijn in zijn/haar medisch vakgebied	14	-0.645
22	Werkzaam als behandelend arts	22	-0.852
19	Getraind of opgeleid in leiderschap	19	-1.183
25	Het belang van de vakgroep centraal stellen	25	-1.235
18	Ervaring in leiderschap	18	-1.470
20	Aanzien bij collega artsen	20	-1.559
26	Een duidelijke functieomschrijving van medisch leiderschap	26	-1.618
21	Een medisch leider ziet zichzelf voornamelijk als arts	21	-1.685

Distinguishing Statements for Factor 1 (P < .05 ; Asterisk (*) Indicates Significance at P < .01)
Both the Factor Q-Sort Value (Q-SV) and the Z-Score (Z-SCR) are Shown.

			Factors					
			1		2		3	
No.	Statement	No.	Q-SV	Z-SCR	Q-SV	Z-SCR	Q-SV	Z-SCR
12	Een duidelijke visie hebben en in staat zijn deze over te br	12	3	1.81*	2	1.14	2	1.22
29	Betrokkenheid bij het vormgeven van de strategie van het zie	29	3	1.51*	-1	-0.79	-1	-0.37
13	PatiÎnt centraal stellen	13	3	1.49	2	1.03	1	0.67
24	Het belang van het gehele ziekenhuis centraal stellen	24	2	1.19*	0	0.26	-2	-1.38
23	In staat om een verbinding te maken tussen het medische -en	23	2	1.06	0	0.23	1	0.56
34	Afdelingsoverstijgende samenwerking opzetten en/of in stand	34	1	0.98*	-1	-1.03	0	0.46
11	Oog hebben voor de kwaliteit en kosten en de balans hierin	11	1	0.76*	0	0.15	0	-0.23
6	In staat zijn om goed samen te werken	6	1	0.61*	3	1.54	2	1.23
1	Goede communicatieve vaardigheden	1	1	0.61*	3	1.72	2	1.14
4	Vaardigheden om een team te managen	4	0	0.04	1	0.54	1	0.61
9	Een team player zijn	9	0	-0.08*	1	0.77	1	0.60
8	Assertiviteit	8	0	-0.31*	1	0.33	-1	-1.10
32	Netwerken en verbindingen maken buiten het ziekenhuis	32	0	-0.35	-1	-0.87	-1	-0.77
27	Acceptatie van de arts als medisch leider	27	-1	-0.50*	-2	-1.11	3	1.23
5	Vaardigheden om een afdeling te managen	5	-1	-0.51	3	1.57	0	-0.10
17	Kennis over het Nederlandse zorgsysteem	17	-1	-0.59	-2	-1.44	0	-0.11
22	Werkzaam als behandelend arts	22	-1	-0.85*	-2	-1.47	1	0.57
26	Een duidelijke functieomschrijving van medisch leiderschap	26	-2	-1.62*	-1	-0.85	3	1.52

Factor Scores -- For Factor 2

No.	Statement	No.	Z-SCORES
1	Goede communicatieve vaardigheden	1	1.721
5	Vaardigheden om een afdeling te managen	5	1.571
6	In staat zijn om goed samen te werken	6	1.544
3	In staat zijn om conflicten op te lossen	3	1.181
12	Een duidelijke visie hebben en in staat zijn deze over te br	12	1.135
10	Integriteit	10	1.051
13	PatiÎnt centraal stellen	13	1.026
2	In staat zijn om anderen te enthousiasmeren en te motiveren	2	1.009
9	Een team player zijn	9	0.765
31	Verbeteringen initiÎren	31	0.663
4	Vaardigheden om een team te managen	4	0.542
19	Getraind of opgeleid in leiderschap	19	0.512
7	Goede onderhandelingsvaardigheden	7	0.376
8	Assertiviteit	8	0.327
24	Het belang van het gehele ziekenhuis centraal stellen	24	0.260
23	In staat om een verbinding te maken tussen het medische -en	23	0.226
28	Voldoende tijd om leiderschapsrol en bijbehorende taken uit	28	0.179
11	Oog hebben voor de kwaliteit en kosten en de balans hierin	11	0.150
33	Verantwoordelijk voor de resultaten van de eenheid waar leid	33	0.040
16	Kennis over structuur en processen van het ziekenhuis	16	-0.131
18	Ervaring in leiderschap	18	-0.149
25	Het belang van de vakgroep centraal stellen	25	-0.513
30	Verantwoordelijk voor het functioneren van medewerkers aan w	30	-0.606
15	Kennis over ziekenhuisfinanciÎn	15	-0.752
29	Betrokkenheid bij het vormgeven van de strategie van het zie	29	-0.786
26	Een duidelijke functieomschrijving van medisch leiderschap	26	-0.852
32	Netwerken en verbindingen maken buiten het ziekenhuis	32	-0.870
14	Excellent zijn in zijn/haar medisch vakgebied	14	-0.990
34	Afdelingsoverstijgende samenwerking opzetten en/of in stand	34	-1.033
27	Acceptatie van de arts als medisch leider	27	-1.105
17	Kennis over het Nederlandse zorgsysteem	17	-1.444
22	Werkzaam als behandelend arts	22	-1.475
21	Een medisch leider ziet zichzelf voornamelijk als arts	21	-1.602
20	Aanzien bij collega artsen	20	-1.972

Distinguishing Statements for Factor 2 (P < .05 ; Asterisk (*) Indicates Significance at P < .01)
Both the Factor Q-Sort Value (Q-SV) and the Z-Score (Z-SCR) are Shown.

			Factors					
			1		2		3	
No.	Statement	No.	Q-SV	Z-SCR	Q-SV	Z-SCR	Q-SV	Z-SCR
1	Goede communicatieve vaardigheden	1	1	0.61	3	1.72	2	1.14
5	Vaardigheden om een afdeling te managen	5	-1	-0.51	3	1.57*	0	-0.10
3	In staat zijn om conflicten op te lossen	3	1	0.27	2	1.18*	0	0.47
19	Getraind of opgeleid in leiderschap	19	-1	-1.18	1	0.51*	-1	-1.02
7	Goede onderhandelingsvaardigheden	7	-1	-0.59	1	0.38*	0	-0.27
8	Assertiviteit	8	0	-0.31	1	0.33*	-1	-1.10
24	Het belang van het gehele ziekenhuis centraal stellen	24	2	1.19	0	0.26*	-2	-1.38
18	Ervaring in leiderschap	18	-2	-1.47	0	-0.15*	-2	-1.36
26	Een duidelijke functieomschrijving van medisch leiderschap	26	-2	-1.62	-1	-0.85*	3	1.52
34	Afdelingsoverstijgende samenwerking opzetten en/of in stand	34	1	0.98	-1	-1.03*	0	0.46
27	Acceptatie van de arts als medisch leider	27	-1	-0.50	-2	-1.11*	3	1.23
17	Kennis over het Nederlandse zorgsysteem	17	-1	-0.59	-2	-1.44*	0	-0.11
22	Werkzaam als behandelend arts	22	-1	-0.85	-2	-1.47*	1	0.57

Factor Scores -- For Factor 3

No.	Statement	No.	Z-SCORES
2	In staat zijn om anderen te enthousiasmeren en te motiveren	2	1.525
26	Een duidelijke functieomschrijving van medisch leiderschap	26	1.525
27	Acceptatie van de arts als medisch leider	27	1.234
6	In staat zijn om goed samen te werken	6	1.233
12	Een duidelijke visie hebben en in staat zijn deze over te br	12	1.224
10	Integriteit	10	1.166
1	Goede communicatieve vaardigheden	1	1.139
28	Voldoende tijd om leiderschapsrol en bijbehorende taken uit	28	0.771
16	Kennis over structuur en processen van het ziekenhuis	16	0.740
13	PatiÎnt centraal stellen	13	0.674
4	Vaardigheden om een team te managen	4	0.610
9	Een team player zijn	9	0.598
22	Werkzaam als behandelend arts	22	0.569
23	In staat om een verbinding te maken tussen het medische -en	23	0.562
3	In staat zijn om conflicten op te lossen	3	0.469
34	Afdelingsoverstijgende samenwerking opzetten en/of in stand	34	0.460
5	Vaardigheden om een afdeling te managen	5	-0.098
17	Kennis over het Nederlandse zorgsysteem	17	-0.110
31	Verbeteringen initiÎren	31	-0.152
11	Oog hebben voor de kwaliteit en kosten en de balans hierin	11	-0.229
7	Goede onderhandelingsvaardigheden	7	-0.267
33	Verantwoordelijk voor de resultaten van de eenheid waar leid	33	-0.310
29	Betrokkenheid bij het vormgeven van de strategie van het zie	29	-0.373
30	Verantwoordelijk voor het functioneren van medewerkers aan w	30	-0.611
21	Een medisch leider ziet zichzelf voornamelijk als arts	21	-0.613
32	Netwerken en verbindingen maken buiten het ziekenhuis	32	-0.765
25	Het belang van de vakgroep centraal stellen	25	-0.912
19	Getraind of opgeleid in leiderschap	19	-1.015
8	Assertiviteit	8	-1.097
18	Ervaring in leiderschap	18	-1.363
24	Het belang van het gehele ziekenhuis centraal stellen	24	-1.379
15	Kennis over ziekenhuisfinanciÎn	15	-1.491
20	Aanzien bij collega artsen	20	-1.674
14	Excellent zijn in zijn/haar medisch vakgebied	14	-2.039

Distinguishing Statements for Factor 3 (P < .05 ; Asterisk (*) Indicates Significance at P < .01)
Both the Factor Q-Sort Value (Q-SV) and the Z-Score (Z-SCR) are Shown.

			Factors					
			1		2		3	
No.	Statement	No.	Q-SV	Z-SCR	Q-SV	Z-SCR	Q-SV	Z-SCR
26	Een duidelijke functieomschrijving van medisch leiderschap	26	-2	-1.62	-1	-0.85	3	1.52*
27	Acceptatie van de arts als medisch leider	27	-1	-0.50	-2	-1.11	3	1.23*
1	Goede communicatieve vaardigheden	1	1	0.61	3	1.72	2	1.14
28	Voldoende tijd om leiderschapsrol en bijbehorende taken uit	28	1	0.32	0	0.18	1	0.77
16	Kennis over structuur en processen van het ziekenhuis	16	0	0.02	0	-0.13	1	0.74*
22	Werkzaam als behandelend arts	22	-1	-0.85	-2	-1.47	1	0.57*
34	Afdelingsoverstijgende samenwerking opzetten en/of in stand	34	1	0.98	-1	-1.03	0	0.46*
5	Vaardigheden om een afdeling te managen	5	-1	-0.51	3	1.57	0	-0.10
17	Kennis over het Nederlandse zorgsysteem	17	-1	-0.59	-2	-1.44	0	-0.11
31	Verbeteringen initiÎren	31	1	0.97	1	0.66	0	-0.15*
21	Een medisch leider ziet zichzelf voornamelijk als arts	21	-3	-1.68	-2	-1.60	-1	-0.61*
8	Assertiviteit	8	0	-0.31	1	0.33	-1	-1.10*
24	Het belang van het gehele ziekenhuis centraal stellen	24	2	1.19	0	0.26	-2	-1.38*
15	Kennis over ziekenhuisfinanciÎn	15	0	-0.42	-1	-0.75	-2	-1.49*
14	Excellent zijn in zijn/haar medisch vakgebied	14	-1	-0.65	-1	-0.99	-3	-2.04*

Consensus Statements -- Those That Do Not Distinguish Between ANY Pair of Factors. All Listed Statements are Non-Significant at P>.01, and Those Flagged With an * are also Non-Significant at P>.05.

			Factors					
			1		2		3	
No.	Statement	No.	Q-SV	Z-SCR	Q-SV	Z-SCR	Q-SV	Z-SCR
2	In staat zijn om anderen te enthousiasmeren en te motiveren	2	2	1.19	1	1.01	3	1.52
10*	Integriteit	10	2	1.38	2	1.05	2	1.17
20	Aanzien bij collega artsen	20	-2	-1.56	-3	-1.97	-2	-1.67
28	Voldoende tijd om leiderschapsrol en bijbehorende taken uit	28	1	0.32	0	0.18	1	0.77
30*	Verantwoordelijk voor het functioneren van medewerkers aan w	30	0	-0.40	-1	-0.61	-1	-0.61
32	Netwerken en verbindingen maken buiten het ziekenhuis	32	0	-0.35	-1	-0.87	-1	-0.77
33*	Verantwoordelijk voor de resultaten van de eenheid waar leid	33	0	-0.21	0	0.04	0	-0.31

Factor Q-Sort Values for Each Statement

No.	Statement	No.	Factor Arrays 1	2	3
1	Goede communicatieve vaardigheden	1	1	3	2
2	In staat zijn om anderen te enthousiasmeren en te motiveren	2	2	1	3
3	In staat zijn om conflicten op te lossen	3	1	2	0
4	Vaardigheden om een team te managen	4	0	1	1
5	Vaardigheden om een afdeling te managen	5	-1	3	0
6	In staat zijn om goed samen te werken	6	1	3	2
7	Goede onderhandelingsvaardigheden	7	-1	1	0
8	Assertiviteit	8	0	1	-1
9	Een team player zijn	9	0	1	1
10	Integriteit	10	2	2	2
11	Oog hebben voor de kwaliteit en kosten en de balans hierin	11	1	0	0
12	Een duidelijke visie hebben en in staat zijn deze over te br	12	3	2	2
13	PatiÎnt centraal stellen	13	3	2	1
14	Excellent zijn in zijn/haar medisch vakgebied	14	-1	-1	-3
15	Kennis over ziekenhuisfinanciÎn	15	0	-1	-2
16	Kennis over structuur en processen van het ziekenhuis	16	0	0	1
17	Kennis over het Nederlandse zorgsysteem	17	-1	-2	0
18	Ervaring in leiderschap	18	-2	0	-2
19	Getraind of opgeleid in leiderschap	19	-1	1	-1
20	Aanzien bij collega artsen	20	-2	-3	-2
21	Een medisch leider ziet zichzelf voornamelijk als arts	21	-3	-2	-1
22	Werkzaam als behandelend arts	22	-1	-2	1
23	In staat om een verbinding te maken tussen het medische -en	23	2	0	1
24	Het belang van het gehele ziekenhuis centraal stellen	24	2	0	-2
25	Het belang van de vakgroep centraal stellen	25	-2	0	-1
26	Een duidelijke functieomschrijving van medisch leiderschap	26	-2	-1	3
27	Acceptatie van de arts als medisch leider	27	-1	-2	3
28	Voldoende tijd om leiderschapsrol en bijbehorende taken uit	28	1	0	1
29	Betrokkenheid bij het vormgeven van de strategie van het zie	29	3	-1	-1
30	Verantwoordelijk voor het functioneren van medewerkers aan w	30	0	-1	-1
31	Verbeteringen initiÎren	31	1	1	0
32	Netwerken en verbindingen maken buiten het ziekenhuis	32	0	-1	-1
33	Verantwoordelijk voor de resultaten van de eenheid waar leid	33	0	0	0
34	Afdelingsoverstijgende samenwerking opzetten en/of in stand	34	1	-1	0
