# Supplementary material for: What makes an ideal hospital-based medical leader? Three views of healthcare professionals and managers: A case study
Source: PLoS One. 2019 Jun 11;14(6):e0218095. doi: 10.1371/journal.pone.0218095 (PMC6559653; doi:10.1371/journal.pone.0218095)
Supplement: S2 File — (PDF) [file pone.0218095.s002.pdf]

S2 File. Statement set and questionnaire.

ENGLISH VERSION

Statement set (all statements were printed on plastic cards):

Personal features

1. Have good communication skills
2. Be able to enthuse and motivate others
3. Be able to resolve conflicts
4. Have the skills to manage a team
5. Have the skills to manage a department
6. Be able to collaborate
7. Have good negotiation skills
8. Be assertive
9. Be a team player
10. Have integrity
11. Have an eye for quality and costs and the balance between them
12. Have a clear vision and be able to convey it to others
13. Be patient centered
14. Be excellent in their medical discipline
15. Knowledge of hospital finances
16. Knowledge of the structure and processes of the hospital
17. Knowledge of the Dutch healthcare system
18. Have experience in leadership
19. Be trained in leadership

20. Be held in high esteem by fellow physicians

21. Consider themselves primarily a physician

22. Be a practicing physician

Context-specific features

23. Be able to connect the clinical and the management domains

24. Focus on the interests of the hospital as a whole

25. Focus on the interests of the clinical departments

26. Have a clear job description of medical leadership

27. Be accepted as a medical leader

28. Have sufficient time to execute the leadership role and all associated tasks

Activities and roles

29. Be involved in strategy development at the hospital level

30. Be responsible for the performance of the employees in his/her department

31. Be able to initiate improvements

32. Network and make alliances outside the hospital

33. Be responsible for the performance of their department

34. Be able to initiate and maintain cross-department collaborations

Instructions participants

1. Please read all factors described on the cards carefully and divide the cards into three piles:
   - Factors that are the most important
   - Factors that are the least important
   - Factors that are neutral important

2. Take the cards from the pile 'most important' and select the two factors which you perceive as the most important for effective medical leadership and place them in the boxes on the right side

of the sheet (under '+3'). There is no difference between the upper or lower box.

3. Select the four factors that are the next most important for effective medical leadership and place them on the sheet (under +2).

4. Repeat this exercise until all cards from the pile 'most important' are placed on the sheet.

5. Repeat step 2 – 4 for the pile 'least important'. Again, start with the two 'least important' factors for effective medical leadership and place the cards on the left side of the sheet (under '-3').

6. Divide the cards of the last pile 'neutral important' over the remaining boxes, according to your preferences.

7. Have a careful look at all boxes and change the order of cards if needed.

Additional questions concerning the Q-sort:

1. Could you explain why these specific two factors on the right side (under +3) are the most important for effective medical leadership according to you?

2. Could you explain why these specific two factors on the left side (under -3) are the least important for effective medical leadership according to you?

3. Could you give specific examples to illustrate why these specific factors are important and others are not?

4. Do you feel that the statement set is complete or are there any factors lacking?

Additional questions

1. Gender (not to be asked)
2. Age
3. Job title
4. Full time or part time
5. Number of years working in current job
6. Number of years working on current department
7. Number of years working in current hospital
8. Management education or courses followed

DUCTH VERSION

Stellingen-set (alle stellingen zijn geprint op plastic kaartjes)

Persoonlijke kenmerken

1. Goede communicatieve vaardigheden
2. In staat zijn om anderen te enthousiasmeren en te motiveren
3. In staat zijn om conflicten op te lossen
4. Vaardigheden om een team te managen
5. Vaardigheden om een afdeling te managen
6. In staat zijn goed samen te werken
7. Goede onderhandelingsvaardigheden
8. Assertiviteit
9. Een team player zijn
10. Integriteit
11. Oog hebben voor de kwaliteit en kosten en de balans hierin
12. Een duidelijke visie hebben en in staat zijn deze over te brengen op anderen
13. Patiënt centraal stellen
14. Excellent zijn in zijn/haar medisch vakgebied
15. Kennis over ziekenhuisfinanciën
16. Kennis over structuur en processen van het ziekenhuis
17. Kennis over het Nederlandse zorgsysteem
18. Ervaring in leiderschap
19. Getraind of opgeleid in leiderschap
20. Aanzien bij collega artsen
21. Een medisch leider ziet zichzelf voornamelijk als arts
22. Werkzaam als behandelend arts

Context specifieke kenmerken

23. In staat om een verbinding te maken tussen het medische- en managementveld
24. Het belang van het gehele ziekenhuis centraal stellen

25. Het belang van de vakgroep centraal stellen
26. Een duidelijke functieomschrijving van medisch leiderschap
27. Acceptatie van de arts als medisch leider
28. Voldoende tijd om leiderschapsrol en bijbehorende taken uit te kunnen voeren

Activiteiten en rollen

29. Betrokkenheid bij het vormgeven van de strategie van het ziekenhuis
30. Verantwoordelijkheid voor het functioneren van medewerkers aan wie leiding wordt gegeven
31. Verbeteringen initiëren
32. Netwerken en verbindingen maken buiten het ziekenhuis
33. Verantwoordelijkheid voor de resultaten van de eenheid waar leiding aan wordt gegeven
34. Afdeling overstijgende samenwerking opzetten en/of in stand houden

Instructies participanten

1. Lees alle factoren op de kaartjes zorgvuldig door en verdeel bij het lezen de kaartjes gelijk in drie stapels:
   - factoren die het **meest belangrijk** zijn (leg deze stapel rechts van u);
   - factoren die het **minst belangrijk** zijn (leg deze stapel links van u);
   - factoren die **neutraal belangrijk** zijn (leg deze stapel in het midden).

2. Neem de kaartjes van de stapel 'meest belangrijk' nog een keer door en selecteer de twee factoren waarvan u vindt dat ze het belangrijkst zijn voor effectief medisch leiderschap en leg deze in de meest rechtse vakken in het schema (onder +3).
   Het maakt niet uit welk kaartje u boven of onder legt!

3. Selecteer vier factoren die daarna het meest belangrijk zijn en leg deze in de vakken onder +2.

4. Herhaal dit tot er geen kaartjes meer over zijn.

5. Herhaal stap 2 t/m 4 ook met de stapel ‘minst belangrijk’. Dus, neem alle kaartjes nog een keer door en selecteer de twee factoren die het minst belangrijk zijn en leg deze in de meest linkse vakken (onder de -3). Selecteer de vier factoren die daarna het minst belangrijk zijn en herhaal dit tot er geen kaartjes meer over zijn.

6. Verdeel de kaartjes van de stapel ‘neutraal belangrijk’ over de overgebleven vakjes, zoals u denkt dat ze moeten liggen.

7. Neem alle kaartjes op het schema door en verplaats kaartjes indien nodig.

Aanvullende vragen betreffende de Q-sort:

1. Kunt u kort toelichten waarom u juist de twee factoren aan het rechter uiteinde (onder +3) het meest belangrijk vindt voor effectief medisch leiderschap?

2. Kunt u toelichten waarom u juist de twee factoren aan het linker uiteinde (onder de -3) het minst belangrijk vindt voor effectief medisch leiderschap?

3. Heeft u concrete voorbeelden ter illustratie waarom deze factoren belangrijk zijn en andere niet.

4. Ontbreken er factoren die bepalen dat sommige medische leiders effectiever zijn dan andere?

Aanvullende vragen:

1. Geslacht (hoeft niet uitgevraagd te worden)
2. Leeftijd
3. Functie
4. Full time/ part time
5. Hoeveel jaar werkzaam in huidige functie
6. Hoeveel jaar werkzaam op huidige afdeling

7. Hoeveel jaar werkzaam in huidige ziekenhuis
8. Management opleiding/cursus
